# Hedonic and Eudaimonic Well-being Across the Lifespan: Differences Between Working and Non-Working Adults

**DOI:** 10.1093/geroni/igaf122.3068

**Published:** 2025-12-31

**Authors:** Piaopiao Cai, Silvia Sörensen

**Affiliations:** University of Rochester, Rochester, New York, United States; University of Rochester Warner School of Education and Human Development, Rochester, New York, United States

## Abstract

The development trajectories of well-being across the lifespan have garnered significant interest in research. Most studies have found evidence of a U-shaped curve trajectory, indicating that midlife adults experience lower well-being compared to younger and older adults across the lifespan. However, the well-being indicators in research have predominantly focused on hedonic aspects, such as hedonic emotions and life satisfaction. The developmental trajectories of eudaimonic well-being, such as six factors of psychological well-being (autonomy, environmental mastery, personal growth, positive relationships, purpose in life, and self-acceptance), have received less attention, particularly concerning the working population. This study utilized a national dataset (n = 2321) to understand the well-being development trajectories between working(n = 1295) and non-working adults (n = 1026) aged 23 to 76. The results of polynomial regressions indicate that only life satisfaction and self-acceptance exhibit a significant quadratic (U-shaped) trend for working and non-working adults. However, the two groups exhibit significantly different ages for minimum life satisfaction (working adults, aged 41.88; non-working adults, aged 37.48) and self-acceptance (working adults, aged 42.98; non-working adults, aged 37.38). Hedonic emotions, autonomy, environmental mastery, and positive relationships, non-working adults exhibit a slightly steeper linear upward trend across the lifespan compared to the linear trajectories of working adults. Additionally, there was a consistently flat linear trajectory for personal growth and purpose in life among both groups. The findings of the present study support Baltes’s (1987) theoretical perspective that individual development is lifelong, multidirectional, and malleable. Further investigation may be needed to better understand the differing trajectories between working and non-working groups.

